# Visuo-haptic multisensory object recognition, categorization, and representation

**DOI:** 10.3389/fpsyg.2014.00730

**Published:** 2014-07-17

**Authors:** Simon Lacey, K. Sathian

**Affiliations:** ^1^Department of Neurology, Emory University School of MedicineAtlanta, GA, USA; ^2^Department of Rehabilitation Medicine, Emory University School of MedicineAtlanta, GA, USA; ^3^Department of Psychology, Emory University School of MedicineAtlanta, GA, USA; ^4^Rehabilitation Research and Development Center of Excellence, Atlanta Veterans Affairs Medical CenterDecatur, GA, USA

**Keywords:** cross-modal, effective connectivity, fMRI, viewpoint dependence, face processing, visual imagery

## Abstract

Visual and haptic unisensory object processing show many similarities in terms of categorization, recognition, and representation. In this review, we discuss how these similarities contribute to multisensory object processing. In particular, we show that similar unisensory visual and haptic representations lead to a shared multisensory representation underlying both cross-modal object recognition and view-independence. This shared representation suggests a common neural substrate and we review several candidate brain regions, previously thought to be specialized for aspects of visual processing, that are now known also to be involved in analogous haptic tasks. Finally, we lay out the evidence for a model of multisensory object recognition in which top-down and bottom-up pathways to the object-selective lateral occipital complex are modulated by object familiarity and individual differences in object and spatial imagery.

## INTRODUCTION

Despite the fact that object perception and recognition are invariably multisensory processes in real life, the haptic modality was for a long time the poor relation in a field dominated by vision science, with the other senses lagging even further behind (Gallace and Spence, 2009; Gallace, 2013). Two things have happened to change this: firstly, from the 1980s, haptics has developed as a field in its own right; secondly, from the 1990s, there has been an accelerated interest in multisensory interactions. Here, we review the interactions and commonalities in visuo-haptic multisensory object processing, beginning with the capabilities and limits of haptic and visuo-haptic recognition. One way to facilitate recognition is to group like objects together: hence, we review recent work on the similarities between visual and haptic categorization and cross-modal transfer of category knowledge. Changes in orientation and size present a major challenge to within-modal object recognition. However, these obstacles seem to be absent in cross-modal recognition and we show that a shared representation underlies both cross-modal recognition and view-independence. We next compare visual and haptic representations from the point of view of individual differences in preferences for object or spatial imagery. A shared representation for vision and touch suggests shared neural processing and therefore we review a number of candidate brain regions, previously thought to be selective for visual aspects of object processing, which have subsequently been shown to be engaged by analogous haptic tasks. This reflects the growing consensus around the concept of a “metamodal” brain with a task-based organization and multisensory inputs, rather than organization around discrete unisensory inputs (Pascual-Leone and Hamilton, 2001; Lacey et al., 2009a; James et al., 2011). Finally, we draw these threads together and discuss the evidence for a model of multisensory visuo-haptic object recognition in which representations are flexibly accessible by either top-down or bottom-up pathways depending on object familiarity and individual differences in imagery preference (Lacey et al., 2009a).

## HAPTIC AND VISUO-HAPTIC OBJECT RECOGNITION

The speed and accuracy of visual object recognition is well-established. Haptic recognition, albeit less well studied, is somewhat slower than visual recognition, but, at least for everyday objects, is still fairly fast and highly accurate with 96% correctly named: 68% in less than 3 s and 94% within 5 s (Klatzky et al., 1985); indeed, a “haptic glance” of less than 1 s suffices in some circumstances (Klatzky and Lederman, 1995). Longer response times in the study of Klatzky et al. (1985) likely reflect the time taken to explore some of the larger items such as a tennis racket or hairdryer. A remarkable fact about haptic processing is that it can be achieved with the feet as well as the hands, albeit more slowly and less accurately, with hand and foot performance being highly correlated across individuals (Lawson, 2014). Haptic identification proceeds, with increasing accuracy, from a “grasp and lift” stage that extracts basic low-level information about a variety of object properties to a series of hand movements that extract more precise information (Klatzky and Lederman, 1992). These hand movements, known as “exploratory procedures,” are property-specific, for example, lateral motion is used to assess texture and contour-following to precisely assess shape (Lederman and Klatzky, 1987). These properties differ in salience to haptic processing depending on the context: under neutral instructions, salience progressively decreases in this order: hardness > texture > shape; under instructions that emphasized haptic processing, the order changes to texture > shape > hardness (Klatzky et al., 1987). Note that the saliency order under neutral instructions is reversed to shape > texture > hardness/size in simultaneous visual and haptic perception, and in haptic perception under instructions to use concurrent visual imagery (Klatzky et al., 1987).

Overall, cross-modal visuo-haptic object recognition, while fairly accurate, comes at a cost compared to within-modal recognition (e.g., Bushnell and Baxt, 1999; Casey and Newell, 2007; and see Lacey et al., 2007). Cross-modal performance is generally better when visual encoding is followed by haptic retrieval than the reverse (e.g., Jones, 1981; Streri and Molina, 1994; Lacey and Campbell, 2006). This asymmetry appears to be a consistent feature of visuo-haptic cross-modal memory but has generally received little attention (e.g., Easton et al., 1997a,b; Reales and Ballesteros, 1999; Nabeta and Kawahara, 2006). One explanation for cross-modal asymmetry might be that shape information is not encoded equally well by the visual and haptic systems, because of competition from other, more salient, modality-specific object properties. Thus, in the haptic-visual cross-modal condition it might be more difficult to encode shape because of the more salient hardness and texture information, as noted above. This effect might be suppressed by the use of concurrent visual imagery in which shape information, common to vision and touch, might be brought to the fore. We should note, however, that when vision and touch are employed simultaneously, properties that are differently weighted in these modalities may be optimally combined on the basis of maximum likelihood estimates (see Ernst and Banks, 2002; Helbig and Ernst, 2007; Helbig et al., 2012; Takahashi and Watt, 2014).

Another explanation for cross-modal asymmetry could be differences in visual and haptic memory capacity. Haptic working memory capacity appears to be limited and variable, and may therefore be more error-prone than visual working memory (Bliss and Hämäläinen, 2005). Alternatively, haptic representations may simply decay faster than visual representations. Rather than a progressive decline over time, the haptic decay function appears to occur entirely in a band of 15–30 s post-stimulus (Kiphart et al., 1992). Consistent with this, a more recent study showed no decline in performance at 15 s (Craddock and Lawson, 2010) although longer intervals were not tested. Haptic-visual performance might therefore be lower because by the time visual recognition is tested, haptically encoded representations have substantially decayed. However, other cross-modal memory studies show that delays up to 30 s (Garvill and Molander, 1973; Woods et al., 2004) or even a week (Pensky et al., 2008) did not affect haptic-visual recognition more than visual-haptic recognition. Thus, an explanation in terms of a simple function of haptic memory properties is likely insufficient.

Cross-modal asymmetry is observed even in very young infants where it is ascribed to constraints imposed by different stages of motor development (Streri and Molina, 1994). But this explanation is also unsatisfactory since the asymmetry persists into maturity (Easton et al., 1997a,b; Bushnell and Baxt, 1999; Lacey and Campbell, 2006). Interestingly, implicit memory does not appear to be affected: cross-modal priming is symmetric (Easton et al., 1997a,b; Reales and Ballesteros, 1999) although verbal encoding strategies may have played a mitigating role in these studies. A recent study suggests that underlying neural activity is asymmetric between the two crossmodal conditions. Using a match-to-sample task, Kassuba et al. (2013) showed that bilateral lateral occipital complex (LOC), fusiform gyrus (FG), and anterior intraparietal sulcus (aIPS) selectively responded more strongly to crossmodal, compared to unimodal, object matching when haptic targets followed visual samples, and more strongly still when the haptic target and visual sample were congruent rather than incongruent; however, these regions showed no such increase for visual targets in either crossmodal or unimodal conditions. This asymmetric increase in activation in the visual-haptic condition may reflect multisensory binding of shape information and suggests that haptics – traditionally seen as the less reliable modality – has to integrate previously presented visual information more than vision has to integrate previous haptic information (Kassuba et al., 2013).

## OBJECT CATEGORIZATION

Categorization facilitates recognition and is critical for much of higher-order cognition (Graf, 2010); hitherto, the emphasis in terms of perceptual categorization has been almost exclusively on the visual, rather than the haptic, modality. More recently, however, a series of studies has systematically compared visual and haptic categorization. Using multi-dimensional scaling analysis, these studies showed that visual and haptic similarity ratings and categorization result in perceptual spaces [i.e., topological representations of the perceived (dis)similarity along a given dimension] that are highly congruent between modalities for novel 3-D objects (Cooke et al., 2007), more realistic 3-D shell-like objects (Gaißert et al., 2008, 2010, 2011) and for natural objects, i.e., actual seashells (Gaißert and Wallraven, 2012). This was so in both unisensory and bisensory conditions (Cooke et al., 2007) and whether 2-D visual objects were compared to haptic 3-D objects (Gaißert et al., 2008, 2010) or passive viewing of 2-D objects was compared to interactive viewing and active haptic exploration of 3-D objects, i.e., such that visual and haptic exploration were more similar (Gaißert et al., 2010). These highly similar visual and haptic perceptual spaces both showed high fidelity to the physical object space [i.e., a topological representation of the actual (dis)similarity along a given dimension; Gaißert et al., 2008, 2010], retaining the category structure (the ordinal adjacency relationships within the category, i.e., the actual progression in variation along a given dimension, for example from roughest to smoothest; Cooke et al., 2007). The isomorphism between perceptual (in either modality) and physical spaces was, furthermore, task-independent, whether simple similarity rating (Gaißert et al., 2008), unconstrained (free sorting), semi-constrained (making exactly three groups) or constrained (matching to a prototype object) categorization (Gaißert et al., 2011). As in vision, haptics also exhibits categorical perception, i.e., discriminability increases sharply when objects belong to different categories and decreases when they belong to the same category (Gaißert et al., 2012).

However, visual and haptic categorization are not entirely alike and, consistent with differential perceptual salience (Klatzky et al., 1987), object properties are differentially weighted depending on the modality, whether they are controlled parametrically (Cooke et al., 2007) or vary naturally (Gaißert and Wallraven, 2012). Shape was more important than texture for visual categorization whereas in haptic and bisensory categorization, shape and texture were approximately equally weighted (Cooke et al., 2007), although in this study shape and texture varied in ways that were intuitive to vision and haptics (broadly, width for shape and smoothness for texture). Using specially manufactured shell-like objects, Gaißert et al. (2010) varied three complex shape parameters that were not intuitive to either modality. While visual and haptic perceptual spaces and the physical object space were all highly similar, the shape dimensions were weighted differently: symmetry was more important than convolutions for vision while the reverse was true for haptics; aperture-tip distance was the least important factor for both modalities (Gaißert et al., 2010). For natural objects – seashells – that varied naturally in a number of properties, similarity ratings and categorization were still driven by global and local shape parameters rather than size, texture, weight etc. (Gaißert and Wallraven, 2012).

These studies suggest a close connection between vision and haptics in terms of similarity mechanisms for categorization but do not necessarily imply a shared representation because of the differential weighting of object properties in each modality. Nonetheless, there is symmetric cross-modal transfer of category information following either visual or haptic category learning, even for complex novel 3-D objects, and furthermore this transfer generalizes to new objects from these categories (Yildirim and Jacobs, 2013). A recent study shows that not only does category membership transfer cross-modally, as shown by Yildirim and Jacobs (2013), but so does category structure (Wallraven et al., 2013), i.e., the ordinal relationships and category boundaries (see Cooke et al., 2007) transcend modality. Crossmodal transfer of category structure is interesting because the ordering of each item within the category is (at least in the studies reviewed here) perceptually driven; thus it may be that a shared multisensory representation underlies cross-modal categorization, as has been suggested for cross-modal recognition (Lacey et al., 2009a; Lacey and Sathian, 2011).

Of course, perceptual similarity is not the only basis for categorization (Smith et al., 1998) and neither vision nor haptics appear to naturally recover categories on alternative bases that are more abstract or semantic. For example, Haag (2011) used realistically textured models of familiar animals that retained real-life size relations, and required visual and haptic categorization on the basis of size (big/small in real life), domesticity (wild/domestic), and predation (carnivore/herbivore). Errors increased as the basis of categorization moved from concrete (size) to abstract (predation) and were consistently greater in haptics than vision (Haag, 2011). Similarly, neither vision nor haptics naturally recovered the taxonomic relationships between the natural seashells used by Gaißert and Wallraven (2012): participants distinguished between concrete categories such as whether the shells used were flat or convoluted, rather than between abstract categories such as gastropods (e.g., sea-snail) vs. bivalves (e.g., oyster). If biological relationships were recovered at all, this was mainly contingent on shape similarities, although vision was better than haptics in this respect (Gaißert and Wallraven, 2012) as it was for the abstract categories studied by Haag (2011).

## FACES: A SPECIAL CATEGORY

Faces are a special category of object that we encounter every day and at which we are especially expert, being able to differentiate large numbers of individuals (Maurer et al., 2002). We are also able to recognize faces under conditions that would impair recognition in other categories; for example, bad lighting or changes in viewpoint (Maurer et al., 2002) – though face recognition is impaired if the face is upside-down (Yin, 1969). An important distinction is made between configural and featural processing: the former refers to processing the spatial relationships between individual facial features as well as the shapes of the features themselves, while the latter refers to the piecemeal processing of individual face parts (Maurer et al., 2002; Dopjans et al., 2012). Although sighted humans obviously recognize faces almost exclusively through vision, live faces can also be identified haptically with high levels of accuracy (over 70%), whether they are learned through touch alone or using both vision and touch (Kilgour and Lederman, 2002). Interestingly, when participants had to haptically identify clay masks produced from live faces, accuracy was significantly lower than for live faces, suggesting that natural material cues and surface properties are important for haptic face recognition (Kilgour and Lederman, 2002). Visual experience may be necessary for haptic face recognition, since the congenitally blind were significantly less accurate than both the sighted and the late-blind (Wallraven and Dopjans, 2013). Nonetheless, haptic face recognition is not as good as visual recognition in the sighted either (Dopjans et al., 2012). This may be due to basic differences between visual and haptic processing. Haptic exploration of any object is almost exclusively sequential and serial (Lederman and Klatzky, 1987; Loomis et al., 1991) whilst visual processing is massively parallel (see Nassi and Callaway, 2009). In the context of face processing, therefore, haptics might be restricted to featural processing, in which individual features are processed independently and have to be assembled into a face context, which may account for lower haptic performance compared to visual configural encoding (Dopjans et al., 2012). When visual encoding was restricted, by using a participant-controlled moving window that only revealed a small portion of the face at a time, so that it was more like haptic sequential processing, visual and haptic performance were more equal (Dopjans et al., 2012), suggesting that any differences arise from different encoding strategies ^1^.

Despite these various differences in performance, visual and haptic face processing do have common aspects. For example, consistent with the shared perceptual spaces discussed above (e.g., Gaißert et al., 2008, 2010, 2011; Gaißert and Wallraven, 2012), there is evidence for similar “face-spaces” for vision and touch in which, again, different properties carry different weights depending on the modality (Wallraven, 2014). The evidence for a face-inversion effect – better recognition when faces are upright than inverted, an effect not seen for non-face categories – is clear for vision but less so for haptics. Kilgour and Lederman (2006) showed a clear haptic inversion effect for faces compared to non-face stimuli, whereas Dopjans et al. (2012) found an inversion effect for unrestricted visual, but not for haptic or restricted visual, face encoding. In “face adaptation,” a neutral face is perceived as having the opposite facial expression to a previously perceived face; for example, adaptation to a sad face leads to perception of a happy face upon subsequent presentation of a face with a neutral expression (e.g., Skinner and Benton, 2010). Such an effect is also seen in within-modal haptic adaptation to faces (Matsumiya, 2012) and transfers cross-modally both from vision to touch and *vice versa*, indicating that haptic face-related information and visual face processing share some common processing (Matsumiya, 2013).

Faces can also be recognized cross-modally between vision and touch (Kilgour and Lederman, 2002); this comes at a cost relative to within-modal recognition (Casey and Newell, 2007) although the cost decreases with familiarity (Casey and Newell, 2005). However, this disadvantage for cross-modal face recognition is unrelated to the encoding modality or to differences in encoding strategies, which suggests that, in contrast to object recognition (see below), vision and touch do not share a common face representation (Casey and Newell, 2007). On the other hand, visually presented faces disrupt identification of haptic faces when their facial expressions are incongruent and facilitate identification when they are congruent (Klatzky et al., 2011) which suggests a shared representation although response competition cannot be excluded as an explanation for these results. However, taken in conjunction with the finding that a visually prosopagnosic patient (i.e., a patient unable to recognize faces visually despite intact basic visual perception) was also unable to recognize faces haptically (Kilgour et al., 2004), a shared representation seems likely.

## OBSTACLES TO EFFICIENT RECOGNITION

### VIEW-DEPENDENCE

**FIGURE 1:**
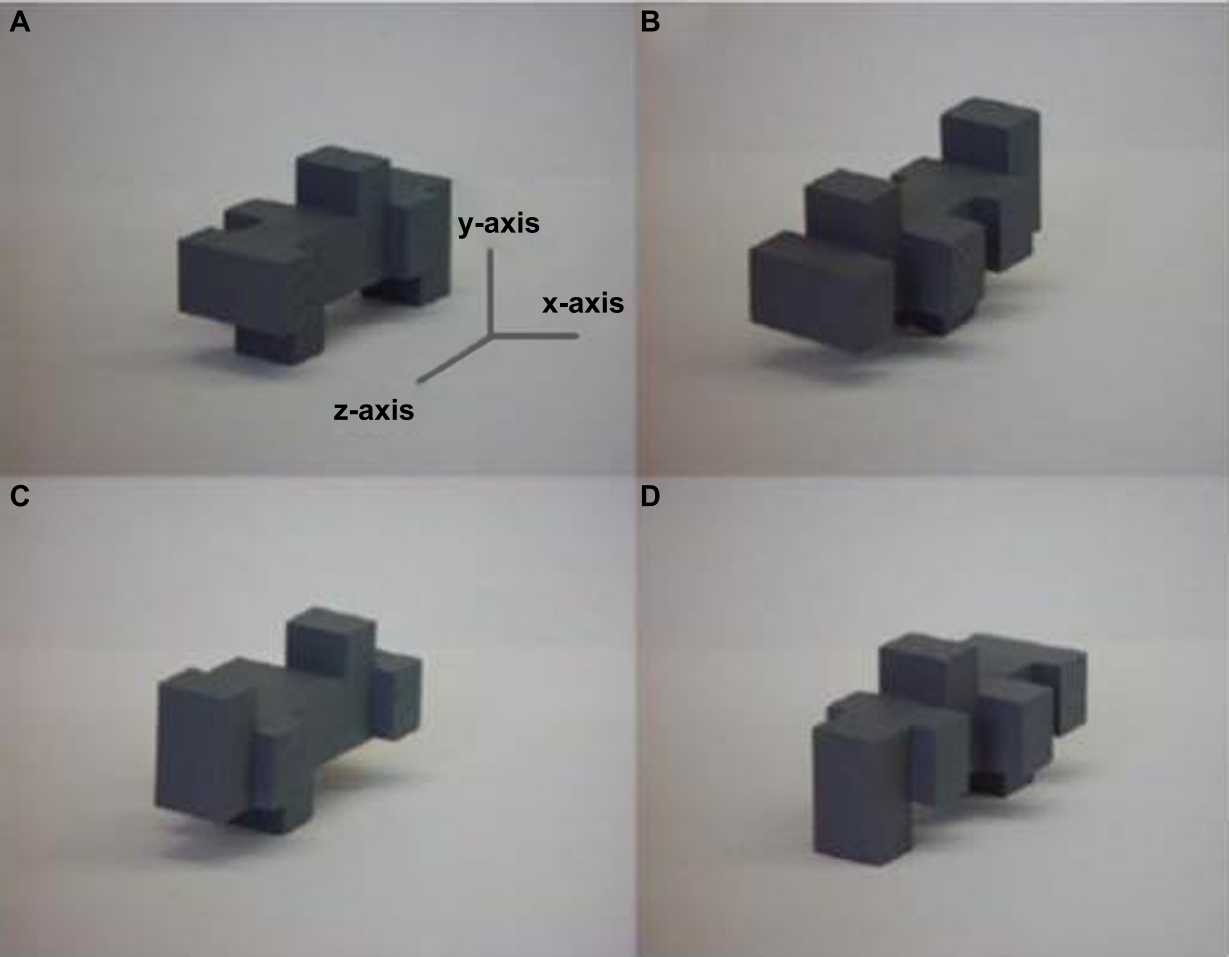
**Example 3-D unfamiliar object shown **(A)** in the original orientation and rotated 180^∘^ about the **(B)** z-axis, **(C)** x-axis, and **(D)** y-axis: rotation about the x- and y-axes are rotations in depth, rotation about the z-axis is a rotation in the picture-plane.** Figure adapted from Lacey et al. (2007).

A change in the orientation of an object changes the related sensory input, e.g., retinal pattern, such that recognition is potentially impaired; an important goal of sensory systems is therefore to achieve perceptual constancy so that objects can be recognized independently of such changes. Visual object recognition is considered view-dependent if rotating an object away from its original orientation impairs subsequent recognition and view-independent if not (reviewed in Peissig and Tarr, 2007). During haptic exploration, the hands can contact an object from different sides simultaneously: intuitively, therefore, one might expect information about several different “views” to be acquired at the same time and that haptic recognition would be view-independent. However, numerous studies have now shown that this intuition is not correct and that haptic object recognition is also view-dependent (Newell et al., 2001; Lacey et al., 2007, 2009b; Ueda and Saiki, 2007, 2012; Craddock and Lawson, 2008, 2010; Lawson, 2009, 2011). The factors underlying haptic view-dependence are not currently known: even unlimited exploration time and orientation cuing do not reduce view-dependence (Lawson, 2011). It is interesting to examine how vision and touch are affected by different types of rotation. Visual recognition is differentially impaired by changes in orientation depending on the axis around which an object is rotated (Gauthier et al., 2002; Lacey et al., 2007). Recognition is slower and less accurate when objects are rotated about the x- and y-axes, i.e., in depth (**Figure 1**), than when rotated about the z-axis, i.e., in the picture plane, for both 2-D (Gauthier et al., 2002) and 3-D stimuli (Lacey et al., 2007). By contrast, haptic recognition is equally impaired by rotation about any axis (Lacey et al., 2007), suggesting that, although vision and haptics are both view-dependent, the basis for this is different in each modality. One possible explanation is that vision and haptics differ in whether or not a surface is occluded by rotation. In vision, a change in orientation can involve not only a transformation in perceptual shape but also occlusion of one or more surfaces – unless the observer physically changes position relative to the object (e.g., Pasqualotto et al., 2005; Pasqualotto and Newell, 2007). Compare, for example, Figures 1A,C – rotation about the x-axis means that the object is turned upside-down and that the former top surface becomes occluded. In haptic exploration, the hands are free to move over all surfaces of an object and to manipulate it into different orientations relative to the hand, thus in any given orientation, no surface is necessarily occluded, provided the object is small enough. If this is true, then no single axis of rotation should be more or less disruptive than another due to surface occlusion, so that haptic recognition only has to deal with a shape transformation. Further work is required to examine whether this explanation is, in fact, correct.

View-dependence mostly occurs when objects are unfamiliar. Increasing object familiarity reduces the disruptive effect of orientation changes and visual recognition tends to become view-independent (Tarr and Pinker, 1989; Bülthoff and Newell, 2006). An exception to this is when a familiar object is typically seen in one specific orientation known as a canonical view, for example the front view of a house (Palmer et al., 1981). View-independence may still occur for a limited range of orientations around the canonical view, but visual recognition is impaired for radically non-canonical views, for example, a teapot seen from directly above (Palmer et al., 1981; Tarr and Pinker, 1989; Bülthoff and Newell, 2006). Object familiarity also results in haptic view-independence and this remains so even where there is a change in the hand used to explore the object (Craddock and Lawson, 2009a). Haptic recognition also reverts to view-dependence for non-canonical orientations (Craddock and Lawson, 2008). However, vision and haptics differ in what constitutes a canonical view. The preferred view in vision is one in which the object is aligned at 45^∘^ to the observer (Palmer et al., 1981) while objects are generally aligned either parallel or orthogonal to the body midline in haptic canonical views (Woods et al., 2008). Canonical views may facilitate view-independent recognition either because they provide the most structural information about an object or because they most closely match a stored representation, but the end result is the same for both vision and haptics (Craddock and Lawson, 2008; Woods et al., 2008).

In contrast to within-modal recognition, visuo-haptic cross-modal recognition is view-independent even for unfamiliar objects that are highly similar (**Figure 1**), whether visual study is followed by haptic test or *vice versa* and whatever the axis of rotation (Lacey et al., 2007, 2010b; Ueda and Saiki, 2007, 2012). Haptic-visual, but not visual-haptic, cross-modal view-independence has been shown for familiar objects (Lawson, 2009). This asymmetry might be due to the fact that the familiar objects used in this particular study were a mixture of scale models (e.g., bed, bath, and shark) and actual-size objects (e.g., jug, pencil); thus, some of these might have been more familiar visually than haptically, resulting in greater error when visually familiar objects had to be recognized by touch. Additional research on the potentially disruptive effects of differential familiarity is merited.

A strange finding is that knowledge of the test modality does not appear to help achieve view-independence. When participants knew the test modality, both visual and haptic within-modal recognition were view-dependent whereas cross-modal recognition was view-independent (Ueda and Saiki, 2007, 2012), but when the test modality was unknown both within- and cross-modal recognition were view-independent (Ueda and Saiki, 2007). At first glance this is puzzling: one would expect that knowledge of the test modality would confer an advantage. However, Ueda and Saiki (2012) showed that eye movements differed during encoding, with longer and more diffuse fixations when participants knew that they would be tested cross-modally (visual-haptic only) compared to within-modally. It is possible that, on the “principle of least commitment” (Marr, 1976), the same pattern of eye movements occurs when the test modality is not known (i.e., it is not possible to commit to an outcome), preserving as much information as possible and resulting in both within- and cross-modal view-independence. Further examination of eye movements during both cross-modal conditions would be valuable, as eye movements could serve as behavioral markers for the multisensory view-independent representation discussed next.

**FIGURE 2:**
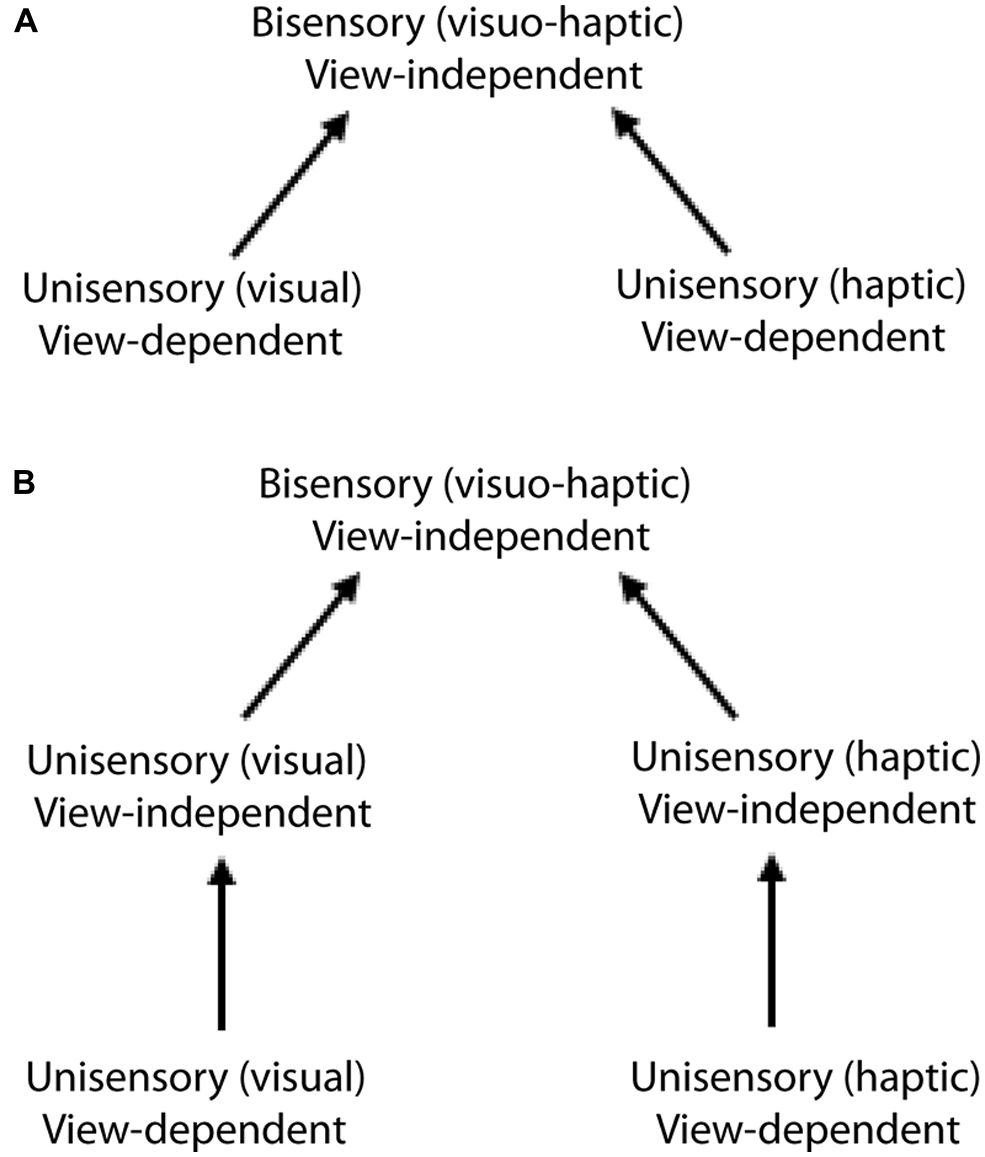
**Alternative models of visuo-haptic view-independence:**(A)** direct integration of the unisensory view-dependent representation into a multisensory view-independent representation; **(B)** bisensory view-independence gated by separate, unisensory view-independent representations.** Evidence supports the direct integration model **(A)**. Figure adapted from Lacey et al. (2009b).

The simplest way in which cross-modal view-independence could arise is that the view-dependent visual and haptic unisensory representations are directly integrated into a view-independent multisensory representation (**Figure 2A**). An alternative explanation is that unisensory view-independence in vision and haptics is a precondition for cross-modal view-independence (**Figure 2B**). In a perceptual learning study, view-independence acquired by learning in one modality transferred completely and symmetrically to the other; thus, whether visual or haptic, within-modal view-independence relies on a single view-independent representation (Lacey et al., 2009b). Furthermore, both visual and haptic within-modal view-independence were acquired following cross-modal training (whether haptic-visual or visual-haptic); we therefore concluded that visuo-haptic view-independence is supported by a single multisensory representation that directly integrates the unisensory view-dependent representations (Lacey et al., 2009b; **Figure 2A**), similar to models that have been proposed for vision (Riesenhuber and Poggio, 1999). Thus, the same representation appears to support both cross-modal recognition and view-independence (whether within- or cross-modal).

### SIZE-DEPENDENCE

In addition to achieving object constancy across orientation changes, the visual system also has to contend with variations in the size of the retinal image that arise from changes in object-observer distance: the same object can produce retinal images that vary in size depending on whether it is near to, or far from, the observer. Presumably, this is compensated by cues arising from depth or motion perception, accounting for the fact that a change in size does not disrupt visual object identification (Biederman and Cooper, 1992; Uttl et al., 2007). However, size change does produce a cost in visual recognition for both unfamiliar (Jolicoeur, 1987) and familiar objects (Jolicoeur, 1987; Uttl et al., 2007). Interestingly, changes in retinal size due to movement of the observer result in better size-constancy than those due to movement of the object (Combe and Wexler, 2010).

Haptic size perception requires integration of both cutaneous (contact area and force) and proprioceptive (finger spread and position) information at initial contact (Berryman et al., 2006). Neither gripping an object tighter, which increases contact area, nor enlarging the spread of the fingers leads us to perceive a change in size (Berryman et al., 2006). Thus, in contrast to vision where perceived size varies with distance, in touch, physical size is perceived directly, i.e., haptic size equals physical size. It is intriguing then, that haptic (Craddock and Lawson, 2009b,c) and cross-modal (Craddock and Lawson, 2009c) recognition are apparently size-dependent and this merits further investigation. Further research should address whether haptic representations store a canonical size for familiar objects (as has recently been proposed for visual representations, Konkle and Oliva, 2011), deviations from which could impair recognition, and whether object constancy can be achieved across size changes in unfamiliar objects.

## REPRESENTATIONS AND INDIVIDUAL DIFFERENCES

A crucial question for object recognition is what information is contained in the mental representations that support it. Visual shape, color, and texture are processed in different cerebral cortical areas (Cant and Goodale, 2007; Cant et al., 2009) but these structural (shape) and surface (color, texture, etc.) properties are integrated in visual object representations (Nicholson and Humphrey, 2003). Changing the color of an object or its part-color combinations between study and test impaired shape recognition, while altering the background color against which objects were presented did not (Nicholson and Humphrey, 2003). This effect could therefore be isolated to the object representation, indicating that this contains both shape and color information (Nicholson and Humphrey, 2003). Visual and haptic within-modal object discrimination are similarly impaired by a change in surface texture (Lacey et al., 2010b), showing firstly that haptic representations also integrate structural and surface properties and secondly that information about surface properties in visual representations is not limited to modality-specific properties like color. In order to investigate whether surface properties are integrated into the multisensory representation underlying cross-modal object discrimination, we tested object discrimination across changes in orientation (thus requiring access to the view-independent multisensory representation discussed above), texture or both. In line with earlier findings (Lacey et al., 2007; Ueda and Saiki, 2007, 2012), cross-modal object discrimination was view-independent when texture did not change; but if texture did change, performance was reduced to chance levels, whether orientation also changed or not (Lacey et al., 2010b). However, some participants were more affected by the texture changes than others. We wondered whether this arose from individual differences in the nature of object representations, which can be conveniently indexed by preferences for different kinds of imagery.

**FIGURE 3:**
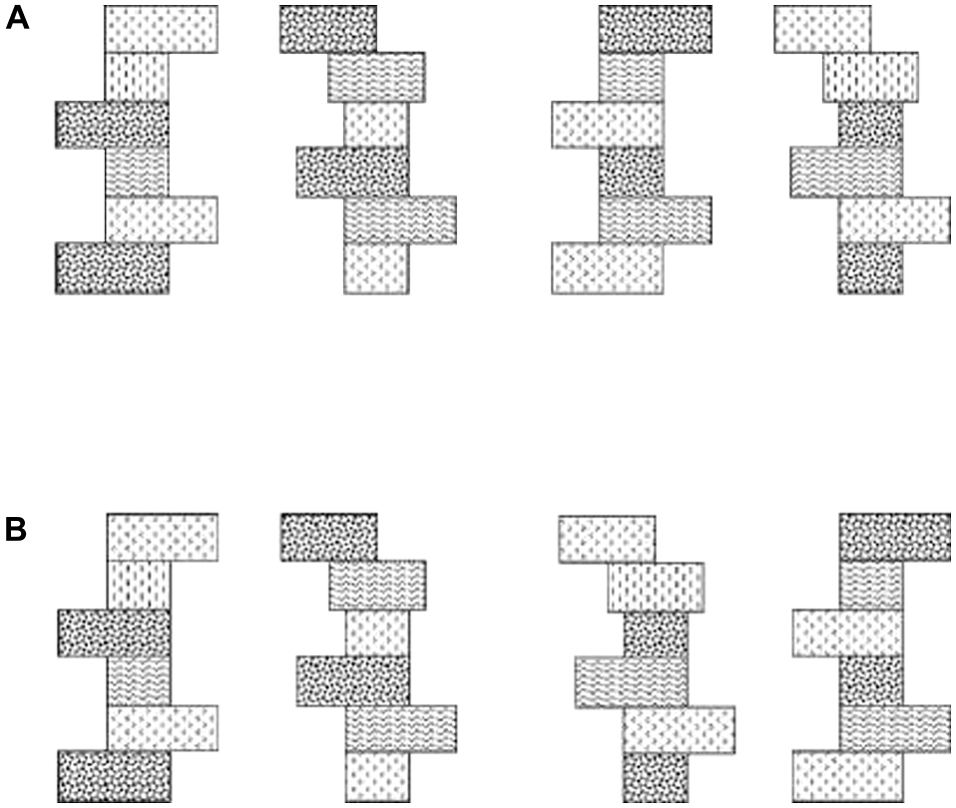
**(A)** Schematic example of Shapes 1 and 2 with (left pair) original texture schemes and (right pair) the texture schemes exchanged. **(B)** Example of Textures 1 and 2 with (left pair) original shapes and (right pair) the shapes exchanged. Figure adapted from Lacey et al. (2011).

Two kinds of visual imagery have been described: “object imagery” (involving pictorial images that are vivid and detailed, dealing with the literal appearance of objects in terms of shape, color, brightness, etc.) and “spatial imagery” (involving schematic images more concerned with the spatial relations of objects, their component parts, and spatial transformations; Kozhevnikov et al., 2002, 2005; Blajenkova et al., 2006). An experimentally important difference is that object imagery includes surface property information while spatial imagery does not. To establish whether object and spatial imagery differences occur in touch as well as vision, we required participants to discriminate shape across changes in texture, and texture across changes in shape (**Figure 3**), in both visual and haptic within-modal conditions. We found that spatial imagers could discriminate shape despite changes in texture but not *vice versa*, presumably because their images tend not to encode surface properties. By contrast, object imagers could discriminate texture despite changes in shape, but not the reverse (Lacey et al., 2011), indicating that texture, a surface property, is integrated into their shape representations. Importantly, visual and haptic performance was not significantly different on either task and performance largely reflected both self-reports of imagery preference and scores on the Object and Spatial Imagery Questionnaire (OSIQ: Blajenkova et al., 2006). Thus, the object-spatial imagery continuum characterizes haptics as well as vision, and individual differences in imagery preference along this continuum affect the extent to which surface properties are integrated into object representations (Lacey et al., 2011). Further analysis of the texture-change condition in our earlier study (Lacey et al., 2010b) showed that performance was indeed related to imagery preference: both object and spatial imagers showed cross-modal view-independence but object imagers were impaired by texture changes whereas spatial imagers were not (Lacey et al., 2011). In addition, the extent of the impairment was correlated with OSIQ scores such that greater preference for object imagery was associated with greater impairment by texture changes; surface properties are therefore likely only integrated into the multisensory representation by object imagers (Lacey et al., 2011). Moreover, spatial imagery preference correlated with the accuracy of cross-modal object recognition (Lacey et al., 2007). It appears, then, that the multisensory representation has some features that are stable across individuals, like view-independence, and some that vary across individuals, such as integration of surface property information and individual differences in imagery preference.

## THE NEURAL BASIS OF VISUO-HAPTIC OBJECT PROCESSING

### SEGREGATED VENTRAL “WHAT” AND DORSAL “WHERE/HOW” PATHWAYS

At the macro-level, visual object processing divides along a ventral pathway concerned with object identity and perception for recognition, and a dorsal pathway dealing with object location and perception for action, e.g., reaching and grasping, (Ungerleider and Mishkin, 1982; Goodale and Milner, 1992). Similar ventral and dorsal pathways have been proposed for the auditory (e.g., De Santis et al., 2007a) and somatosensory domains (Dijkerman and de Haan, 2007), with divergence of the “what” and “where/how” pathways in a similar timeframe (∼200 ms after stimulus onset) (De Santis et al., 2007a,b), and thus are probably common aspects of functional architecture across modalities.

In the case of touch, an early functional magnetic resonance (fMRI) study found that haptic object recognition activated frontal cortical areas as well as inferior parietal cortex, while a haptic object location task activated superior parietal regions (Reed et al., 2005). A later study from our laboratory (Sathian et al., 2011) compared perception of haptic texture and location, reasoning that texture would be a better marker of haptic object identity, given the salience of texture to touch (Klatzky et al., 1987). This study found that, while both visual and haptic location judgments involved a similar dorsal pathway comprising large sectors of the IPS and frontal eye fields (FEFs) bilaterally, haptic texture perception engaged extensive areas of the parietal operculum (OP), which contains higher-order (i.e., non-primary), ventral regions of somatosensory cortex. In addition, shared cortical processing of texture across vision and touch was found in parts of extrastriate (i.e., non-primary) visual cortex and ventral premotor cortex (Sathian et al., 2011). For both texture and location, several of these bisensory areas showed correlations of activation magnitude between the visual and haptic tasks, indicating some commonality of cortical processing across modalities (Sathian et al., 2011). Another group extended these findings by showing that early visual cortex showed activation magnitudes that not only scaled with the interdot spacing of dot-patterns, but were also modulated by the presence of matching haptic input (Eck et al., 2013).

### MULTISENSORY PROCESSING OF OBJECT SHAPE

Cortical areas in both the ventral and dorsal pathways previously identified as specialized for various aspects of visual processing are also functionally involved during the corresponding haptic tasks (for reviews see Amedi et al., 2005; Sathian and Lacey, 2007; Lacey and Sathian, 2011). In the human visual pathway even early visual areas (which project to both dorsal and ventral streams) have been found to respond to changes in haptic shape, suggesting that haptic shape perception might involve the entire ventral stream (Snow et al., 2014). If true, this might reflect cortical pathways between primary somatosensory and visual cortices previously demonstrated in the macaque (Négyessy et al., 2006); however, as with other studies (see below), it is not possible to exclude visual imagery as an explanation for the findings of Snow et al. (2014). The majority of research on visuo-haptic processing of object shape has concentrated on higher-level visual areas, in particular the LOC, an object-selective region in the ventral visual pathway (Malach et al., 1995), a sub-region of which also responds selectively to objects in both vision and touch (Amedi et al., 2001, 2002; Stilla and Sathian, 2008). The LOC responds to both haptic 3-D (Amedi et al., 2001; Zhang et al., 2004; Stilla and Sathian, 2008) and tactile 2-D stimuli (Stoesz et al., 2003; Prather et al., 2004) but does not respond during auditory object recognition cued by object-specific sounds (Amedi et al., 2002). However, when participants listened to the impact sounds made by rods and balls made of either metal or wood and categorized these sounds by the shape of the object that made them, the material of the object, or by using all the acoustic information available, the LOC was more activated when these sounds were categorized by shape than by material (James et al., 2011). Here again though, participants could have solved this matching task using visual imagery: we return to the potential role of visual imagery in a later section.

The LOC does, however, respond to auditory shape information created by a visual-auditory sensory substitution device (Amedi et al., 2007) using a specific algorithm to convert visual information into an auditory stream or “soundscape” in which the visual horizontal axis is represented by auditory duration and stereo panning, the visual vertical axis by variations in tone frequency, and pixel brightness by variations in tone loudness. Although it requires extensive training, both sighted and blind humans can learn to recognize objects by extracting shape information from such soundscapes (Amedi et al., 2007). However, the LOC only responds to soundscapes created according to the algorithm – and which therefore represent shape in a principled way – and not when participants learn soundscapes that are merely arbitrarily associated with particular objects (Amedi et al., 2007). Thus, the LOC can be regarded as processing geometric shape information independently of the sensory modality used to acquire it.

Apart from the LOC, multisensory (visuo-haptic) responses have also been observed in several parietal regions: in particular, the aIPS is involved in perception of both the shape and location of objects, with co-activation of the LOC for shape and the FEF for location (Stilla and Sathian, 2008; Sathian et al., 2011; see also Saito et al., 2003). The postcentral sulcus (PCS; Stilla and Sathian, 2008), corresponding to Brodmann’s area 2 of primary somatosensory cortex (S1; Grefkes et al., 2001), also shows visuo-haptic shape-selectivity. This area is normally considered exclusively somatosensory but the bisensory responses observed by Stilla and Sathian (2008) are consistent with earlier neurophysiological studies that suggested visual responsiveness in parts of S1 (Iriki et al., 1996; Zhou and Fuster, 1997).

Multisensory responses in the LOC and elsewhere might reflect visuo-haptic integration in neurons that process both visual and haptic input; alternatively, they might arise from separate inputs to discrete but interdigitated unisensory neuronal populations. Tal and Amedi (2009) sought to distinguish between these using fMRI adaptation (fMR-A). This technique utilizes the repetition suppression effect, i.e., when the same stimulus is repeated, the blood-oxygen level dependent (BOLD) signal is attenuated. Since repetition suppression can be observed in single neurons, fMR-A can reveal neuronal selectivity profiles (see Grill-Spector et al., 2006; Krekelberg et al., 2006 for reviews). When stimuli that had been presented visually were presented again haptically, there was a robust cross-modal adaptation effect not only in the LOC and the aIPS, but also in bilateral precentral sulcus (preCS) corresponding to ventral premotor cortex, and the right anterior insula, suggesting that these areas were integrating multisensory inputs at the neuronal level. However, a separate preCS site and posterior parts of the IPS did not show cross-modal adaptation, suggesting that their multisensory responses arise from separate unisensory populations. Because fMR-A effects may not necessarily reflect neuronal selectivity (Mur et al., 2010), it will be necessary to confirm the findings of Tal and Amedi (2009) with converging evidence using other methods.

It is critical to determine whether haptic or tactile involvement in supposedly visual cortical areas is functionally relevant, i.e., whether it is actually necessary for task performance. Although research along these lines is still relatively sparse, two lines of evidence indicate that this is indeed the case. Firstly, case studies indicate that the LOC is necessary for both haptic and visual shape perception. A lesion to the left occipito-temporal cortex, which likely included the LOC, resulted in both tactile and visual agnosia even though somatosensory cortex and basic somatosensory function were intact (Feinberg et al., 1986). Another patient with bilateral LOC lesions was unable to learn new objects either visually or haptically (James et al., 2006b). These case studies are consistent with the existence of a shared multisensory representation in the LOC.

Transcranial magnetic stimulation (TMS) is a technique used to temporarily deactivate specific, functionally defined, cortical areas, i.e., to create “virtual lesions” (Sack, 2006). TMS over a parieto-occipital region previously shown to be active during tactile grating orientation discrimination (Sathian et al., 1997) interfered with performance of this task (Zangaladze et al., 1999) indicating that it was functionally, rather than epiphenomenally, involved. This area is the probable human homolog of macaque area V6 (Pitzalis et al., 2006). Repetitive TMS (rTMS) over the left LOC impaired visual object, but not scene, categorization (Mullin and Steeves, 2011), similarly suggesting that this area is necessary for object processing. rTMS over the left aIPS impaired visual-haptic, but not haptic-visual, shape matching using the right hand (Buelte et al., 2008), but shape matching with the left hand during rTMS over the right aIPS was unaffected in either cross-modal condition. The reason for this discrepancy is unclear, and emphasizes that the precise roles of the IPS and LOC in multisensory shape processing have yet to be fully worked out.

### CATEGORY-SPECIFIC REPRESENTATIONS

There has been rather limited neural study of cross-modal category-selective representations. Using multivoxel pattern analysis of fMRI data, Pietrini et al. (2004) demonstrated that selectivity for particular categories of man-made objects was correlated across vision and touch in a region of inferotemporal cortex. In the case of face perception, fMRI studies, in contrast to the behavioral studies reviewed above, tend to favor separate, rather than shared representations. For example, visual and haptic face-selectivity in ventral and inferior temporal cortex are in largely separate voxel populations (Pietrini et al., 2004). Haptic face recognition activates the left FG, whereas visual face recognition activates the right FG (Kilgour et al., 2005); furthermore, activity in the left FG increases during haptic processing of familiar, compared to unfamiliar, faces while the right FG remains relatively inactive (James et al., 2006a). A further difference in FG face responses is that imagery of visually presented faces activates the left FG more than the right FG (Ishai et al., 2002) ^2^; this raises the possibility that haptic face perception involves visual imagery mechanisms. Although one study found that haptic face recognition ability and imagery vividness ratings were uncorrelated (Kilgour and Lederman, 2002), the implication of visual imagery in haptic face perception is very consonant with our findings in haptic shape perception discussed below (Deshpande et al., 2010; Lacey et al., 2010a) especially as vividness ratings do not particularly index imagery ability (reviewed in Lacey and Lawson, 2013). Further studies are needed to resolve the neural basis of multisensory face perception, and its differences from multisensory object perception.

### VIEW- AND SIZE-INDEPENDENCE

The cortical locus of the multisensory view-independent representation is currently not known. Evidence for visual view-independence in the LOC is mixed: as might be expected, unfamiliar objects produce view-dependent LOC responses (Gauthier et al., 2002) and familiar objects produce view-independent responses (Valyear et al., 2006; Eger et al., 2008a; Pourtois et al., 2009). By contrast, one study found view-dependence in the LOC even for familiar objects, although in this study there was position-independence (Grill-Spector et al., 1999), whereas another found view-independence for both familiar and unfamiliar objects (James et al., 2002a). A recent TMS study of 2-D shape suggests that the LOC is functionally involved in view-independent recognition (Silvanto et al., 2010) but only two rotations, 20 and 70^∘^, were tested and TMS effects were only seen for the 20^∘^ rotation; further work is required to substantiate this finding. Responses in the FG are also variable with the left FG less sensitive to orientation changes than the right FG (Andresen et al., 2009; Harvey and Burgund, 2012). A study of face viewpoint-selectivity showed a gradient of decreasing orientation sensitivity, from view-dependence in early visual cortex to partial view-independence in later areas including LOC (Axelrod and Yovel, 2012); this sensitivity gradient may also apply to non-face objects.

Various parietal regions show visual view-dependent responses, e.g., the IPS (James et al., 2002a) and a parieto-occipital area (Valyear et al., 2006). Superior parietal cortex is view-dependent during mental rotation but not visual object recognition (Gauthier et al., 2002; Wilson and Farah, 2006). As these regions are in the dorsal pathway, concerned with object location and perception for action, view-dependent responses in these regions are not surprising (Ungerleider and Mishkin, 1982; Goodale and Milner, 1992). Actions such as reaching and grasping adapt to changes in object orientation and consistent with this, lateral parieto-occipital cortex shows view-dependent responses for graspable, but not for non-graspable objects (Rice et al., 2007).

To date, we are not aware of neuroimaging studies of haptic or cross-modal processing of stimuli across changes in orientation. James et al. (2002b) varied object orientation, but this study concentrated on haptic-to-visual priming rather than the cross-modal response to same vs. different orientations *per se*. Additionally, there is much work to be done on the effect of orientation changes when shape information is derived from the auditory soundscapes produced by sensory substitution devices (SSDs) and also when the options for haptically interacting with an object are altered by a change in orientation. Similarly, there is no neuroimaging work on haptic and multisensory processing of stimuli across changes in size. However, visual size-independence has been consistently observed in the LOC (Grill-Spector et al., 1999; Ewbank et al., 2005; Eger et al., 2008a,b), with anterior regions showing more size-independence than posterior regions (Sawamura et al., 2005; Eger et al., 2008b).

**FIGURE 4:**
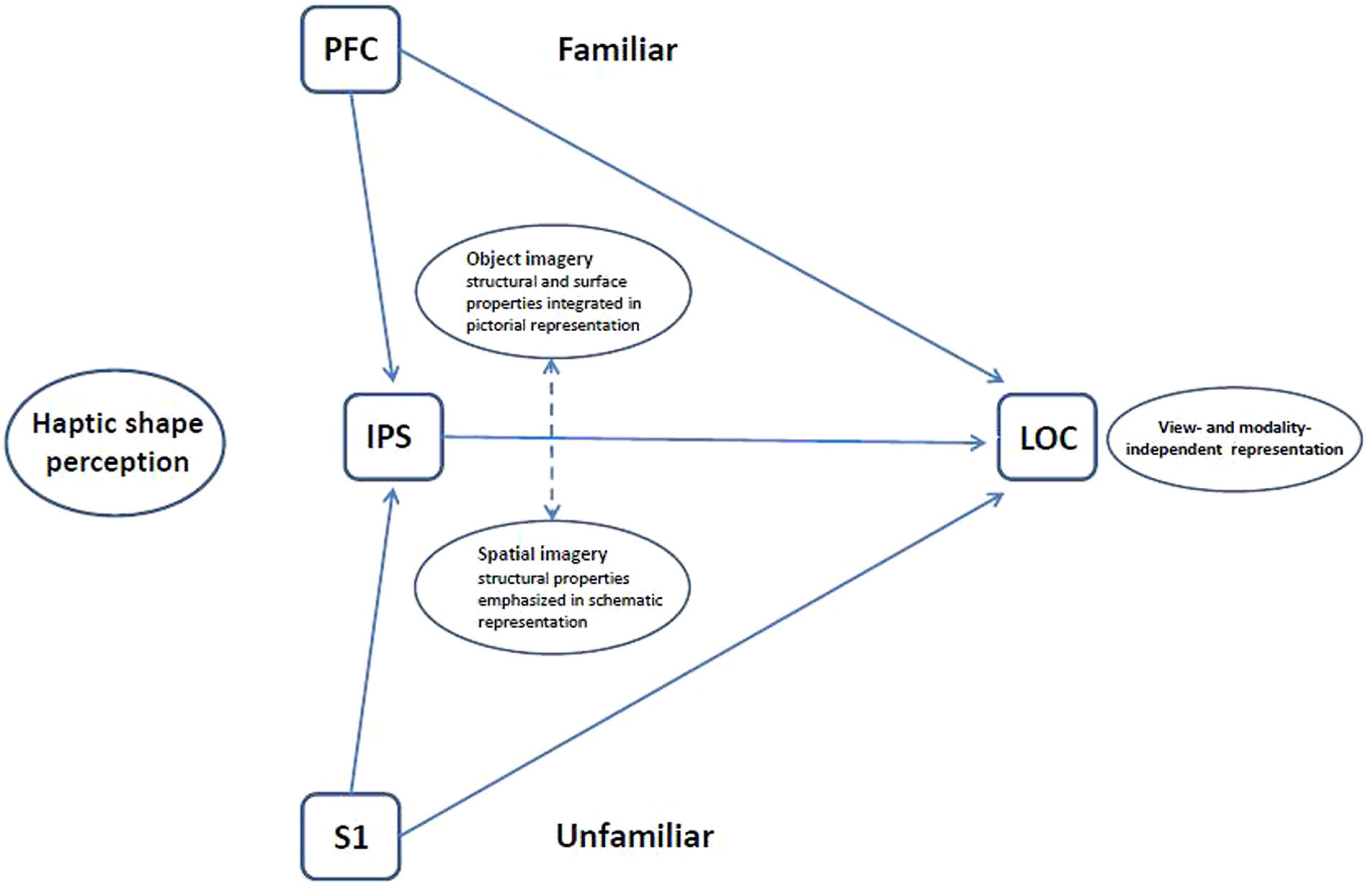
**Schematic model of haptic object representation in LOC modulated by object familiarity and imagery type.** For unfamiliar more than familiar objects, the LOC is driven bottom-up from somatosensory cortex (S1) with support from spatial imagery processes in the IPS. For familiar more than unfamiliar objects, the LOC is driven top-down from prefrontal cortex (PFC) via object imagery processes. The LOC thus houses an object representation that is flexibly accessible, both bottom-up and top-down, and which is modality- and view-independent (Lacey et al., 2007, 2009b, 2011).

## A MODEL OF VISUO-HAPTIC MULTISENSORY OBJECT REPRESENTATION

Haptic activation of the LOC might arise from direct somatosensory input. Activity in somatosensory cortex propagates to the LOC as early as 150 ms after stimulus onset during tactile discrimination of simple shapes, a timeframe consistent with “bottom-up” projections to LOC (Lucan et al., 2010; Adhikari et al., 2014). Similarly, in a tactile microspatial discrimination task, LOC activity was consistent with feedforward propagation in a beta-band oscillatory network (Adhikari et al., 2014). In addition, a patient with bilateral ventral occipito-temporal lesions, but with sparing of the dorsal part of the LOC that likely included the multisensory sub-region, showed visual agnosia but intact haptic object recognition (Allen and Humphreys, 2009). Haptic object recognition was associated with activation of the intact dorsal part of the LOC, suggesting that somatosensory input could directly activate this region (Allen and Humphreys, 2009).

Alternatively, haptic perception might evoke visual imagery of the felt object resulting in “top-down” activation of the LOC (Sathian et al., 1997) and consistent with this hypothesis, many studies show LOC activity during visual imagery. During auditorily cued mental imagery of familiar object shape, both blind and sighted participants show left LOC activation, where shape information would arise mainly from haptic experience for the blind and mainly from visual experience for the sighted (De Volder et al., 2001). The left LOC is also active when geometric and material object properties are retrieved from memory (Newman et al., 2005) and haptic shape-selective activation magnitudes in the right LOC were highly correlated with ratings of visual imagery vividness (Zhang et al., 2004). A counter-argument is that imagery plays a relatively minor role because LOC activity was substantially lower during visual imagery compared to haptic shape perception (Amedi et al., 2001). However, this study could not verify that participants engaged in imagery throughout the imaging session, so that lower imagery-related activity might have resulted from non-compliance (or irregular compliance) with the task. It has also been argued that visual imagery cannot explain haptically evoked LOC activity because early- as well as late-blind individuals show shape-related LOC activation via both touch (reviewed in Pascual-Leone et al., 2005; Sathian, 2005; Sathian and Lacey, 2007) and hearing using SSDs (Arno et al., 2001; Renier et al., 2004, 2005; Amedi et al., 2007). But this argument, while true for the early blind, does not rule out a visual imagery explanation in the sighted, given the extensive evidence for cross-modal plasticity following visual deprivation (reviewed in Pascual-Leone et al., 2005; Sathian, 2005; Sathian and Lacey, 2007).

In this section we describe a model of visuo-haptic multisensory object representation (Lacey et al., 2009a) and review the evidence for this model from studies designed to explicitly test the visual imagery hypothesis discussed above (Deshpande et al., 2010; Lacey et al., 2010a, 2014). In this model, object representations in the LOC can be flexibly accessed either bottom-up or top-down, depending on object familiarity, and independently of the input modality. There is no stored representation for unfamiliar objects so that during haptic recognition, an unfamiliar object has to be explored in its entirety in order to compute global shape and to relate component parts to one another. This, we propose, occurs in a bottom-up pathway from somatosensory cortex to the LOC, with involvement of the IPS in computing part relationships and thence global shape, facilitated by spatial imagery processes. For familiar objects, global shape can be inferred more easily, perhaps from distinctive features or one diagnostic part, and we suggest that haptic exploration rapidly acquires enough information to trigger a stored visual image and generate a hypothesis about its identity, as has been proposed for vision (e.g., Bar, 2007). This occurs in a top-down pathway from prefrontal cortex to LOC, involving primarily object imagery processes (though spatial imagery may still have a role in processing familiar objects, for example, in view-independent recognition).

We tested this model using analyses of inter-task correlations of activation magnitude between visual object imagery and haptic shape perception (Lacey et al., 2010a) and analyses of effective connectivity (Deshpande et al., 2010), reasoning that reliance on similar processes across tasks would lead to correlations of activation magnitude across participants, as well as similar patterns of effective connectivity across tasks. In contrast to previous studies, we ensured that participants engaged in visual imagery throughout each scan by using an object imagery task and recording responses. Participants also performed a haptic shape discrimination task using either familiar or unfamiliar objects. We found that object familiarity modulated inter-task correlations as predicted by our model. There were eleven regions common to visual object imagery and haptic perception of familiar shape, six of which (including bilateral LOC) showed inter-task correlations of activation magnitude. By contrast, object imagery and haptic perception of unfamiliar shape shared only four regions, only one of which (an IPS region) showed an inter-task correlation (Lacey et al., 2010a). More recently, we examined the relation between haptic shape perception and spatial imagery, using a spatial imagery task in which participants memorized a 4 × 4 lettered grid and, in response to auditory letter strings, constructed novel shapes within the imagined grid from component parts (Lacey et al., 2014); the haptic shape tasks were the same as in Lacey et al. (2010a). Contrary to the model, relatively few regions showed inter-task correlations between spatial imagery and haptic perception of either familiar or unfamiliar shape, with parietal foci featuring in both sets of correlations. This suggests that spatial imagery is relevant to haptic shape perception regardless of object familiarity, whereas our earlier finding suggested that object imagery is more strongly associated with haptic perception of familiar, than unfamiliar, shape (Lacey et al., 2010a). However, it is also possible that the parietal foci showing inter-task correlations between spatial imagery and haptic shape perception reflect spatial processing more generally, rather than spatial imagery *per se* (Lacey et al., 2014; and see Jäncke et al., 2001), or generic imagery processes, e.g., image generation, common to both object and spatial imagery (Lacey et al., 2014; and see Mechelli et al., 2004).

In our study of spatial imagery (Lacey et al., 2014), we also conducted effective connectivity analyses, based on the inferred neuronal activity derived from deconvolving the hemodynamic response out of the observed BOLD signals (Sathian et al., 2013). In order to make direct comparisons between the neural networks underlying object and spatial imagery in haptic shape perception, we re-analyzed our earlier data (Deshpande et al., 2010) using the newer effective connectivity methods. These analyses supported the broad architecture of the model, showing that the spatial imagery network shared much more commonality with the network associated with unfamiliar, compared to familiar, shape perception, while the object imagery network shared much more commonality with familiar, than unfamiliar, shape perception (Lacey et al., 2014). More specifically, the model proposes that the component parts of an unfamiliar object are explored in their entirety and assembled into a representation of global shape via spatial imagery processes (Lacey et al., 2009a). Consistent with this, in the parts of the network that were common to spatial imagery and unfamiliar haptic shape perception, the LOC is driven by parietal foci, with complex cross-talk between posterior parietal and somatosensory foci. These findings fit with the notion of bottom-up pathways from somatosensory cortex and a role for cortex in and around the IPS in spatial imagery (Lacey et al., 2014). The IPS and somatosensory interactions were absent from the sparse network that was shared by spatial imagery and haptic perception of familiar shape. By contrast, the relationship between object imagery and familiar shape perception is characterized by top-down pathways from prefrontal areas reflecting the involvement of object imagery, according to our model (Lacey et al., 2009a). The re-analyzed data supported this, showing the LOC driven bilaterally by the left inferior frontal gyrus in the network shared by object imagery and haptic perception of familiar shape, while these pathways were absent from the extremely sparse network common to object imagery and unfamiliar haptic shape perception (Lacey et al., 2014).

**Figure 4** shows the current version of our model for haptic shape perception in which the LOC is driven bottom-up from primary somatosensory cortex as well as top-down via object imagery processes from prefrontal cortex, with additional input from the IPS involving spatial imagery processes. We propose that the bottom-up route is more important for haptic perception of unfamiliar than familiar objects, whereas the converse is true of the top-down route – more important for haptic perception of familiar than unfamiliar objects. It will be interesting to explore the impact of individual preferences for object vs. spatial imagery on these processes and paths.

## SUMMARY

The research reviewed here illustrates how deeply interconnected the visual and haptic modalities are in object processing, from highly similar and transferable perceptual spaces underlying categorization, through shared representations in cross-modal and view-independent recognition and commonalities in imagery preferences, to multisensory neural substrates and complex interactions between bottom-up and top-down processes as well as between object and spatial imagery. Much, however, remains to be done in order to provide a detailed account of visuo-haptic multisensory behavior and its underlying mechanisms and how this understanding can be put to use, for example in the service of neurorehabilitation, particularly for those with sensory deprivation of various sorts.

## Conflict of Interest Statement

The authors declare that the research was conducted in the absence of any commercial or financial relationships that could be construed as a potential conflict of interest.

## Footnotes

1 For discussions of configural versus featural visual face processing, see Peterson and Rhodes (2003).

2 Note that, although these studies mainly refer to the fusiform gyrus, this is not the only cortical region involved in face processing, nor are faces necessarily the only category processed in that, or other, regions; this issue remains controversial (see Harel et al., 2013, for a review).
